# Teaching an experiential field course via online participatory science projects: A COVID‐19 case study of a UC California Naturalist course

**DOI:** 10.1002/ece3.7187

**Published:** 2021-01-27

**Authors:** Laci M. Gerhart, Christopher C. Jadallah, Sarah S. Angulo, Gregory C. Ira

**Affiliations:** ^1^ University of California Davis CA USA; ^2^ UC California Naturalist Program UC Agriculture and Natural Resources Davis CA USA

**Keywords:** California Naturalist, citizen science, COVID‐19, environmental literacy, field course, natural history, online pedagogy, participatory science, remote instruction, undergraduate education

## Abstract

Experience and training in field work are critical components of undergraduate education in ecology, and many university courses incorporate field‐based or experiential components into the curriculum in order to provide students hands‐on experience. Due to the onset of the COVID‐19 pandemic and the sudden shift to remote instruction in the spring of 2020, many instructors of such courses found themselves struggling to identify strategies for developing rigorous field activities that could be completed online, solo, and from a student's backyard. This case study illustrates the process by which one field‐based course, a UC California Naturalist certification course offered at the University of California, Davis, transitioned to fully remote instruction. The transition relied on established, publicly available, online participatory science platforms (e.g., iNaturalist) to which the students contributed data and field observations remotely. Student feedback on the course and voluntary‐continued engagement with the participatory science platforms indicates that the student perspective of the experience was on par with previous traditional offerings of the course. This case study also includes topics and participatory science resources for consideration by faculty facing a similar transition from group field activities to remote, individual field‐based experiences.

## Graphical abstract

Experience and training in field work is a critical component of undergraduate education in ecology, and many university courses incorporate field‐based or experiential components into the curriculum in order to provide students hands‐on experience. Due to the onset of the COVID‐19 pandemic and the sudden shift to remote instruction in the spring of 2020, many instructors of such courses found themselves struggling to identify rigorous strategies for developing field activities that could be completed online, solo, and from a student’s backyard. This case study illustrates the process by which one field‐based course transitioned to fully remote instruction by relying on established, publicly available, online participatory science platforms (e.g., iNaturalist).

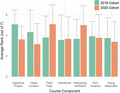

## 1 INTRODUCTION

Fieldwork and field activities are an important component of courses in a variety of fields, including ecology, and environmental science (Fleischner et al., 2017). Formally, ‘fieldwork’ can be defined as “any component of the curriculum that involves leaving the classroom and learning through first‐hand experience” (Boyle et al., 2007) including formal structured research, interpretive hikes, or any other outdoor observation, experience, or activity. In ecology and environmental science, fieldwork components in courses can benefit students through development of technical and transferable skills (Peasland et al., 2019), increased confidence and motivation to learn (Boyle et al., 2007), and increased favorable attitudes toward environmental protection (Fernández Manzanal et al., 1999). Additionally, courses with field activity components have been shown to reduce the achievement and completion gaps in underrepresented groups through increased self‐efficacy, reduced attrition, higher graduation rates, and higher final GPAs (Beltran et al., 2020). Unfortunately, skills relating to natural history and naturalism have been specifically identified as lacking in undergraduate education and in formal training of ecology and environmental science professionals despite their importance in these fields (Barrows et al., 2016). The course described in this paper, and the California Naturalist program as a whole, seeks to fill this gap.

In the spring of 2020, when shelter‐in‐place guidelines were enacted in response to the COVID‐19 pandemic, institutions of higher education were forced to develop strategies for continuing instruction in a fully online and remote environment (Crawford et al., 2020). The impacts of the pandemic on teaching and learning in higher education are still being understood and indeed form the basis of this special issue (Lashley et al., 2020). Programs with an emphasis on fieldwork, applied training, and/or practical experience were particularly impacted (Ahmed et al., 2020; Corlett et al., 2020). Courses in these programs were therefore faced with a conundrum: how to incorporate the educational and experiential value of fieldwork components into remote online instruction. This case study follows the transition from in‐person (‘traditional’) to remote instruction of a UC California Naturalist (CalNat) certification course, Wild Davis, offered at the University of California, Davis (UCD).

**FIGURE 1:**
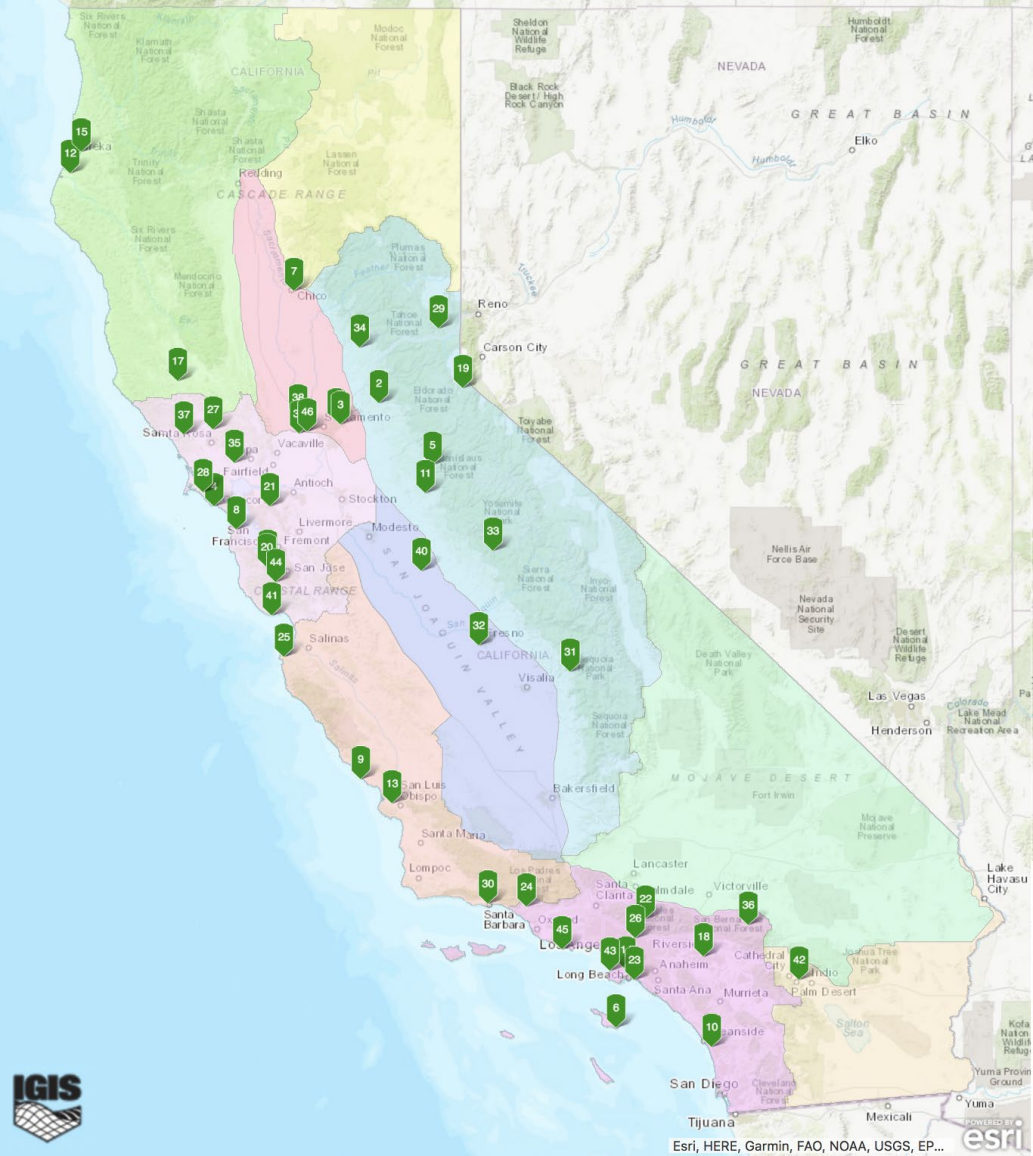
Map of CalNat courses across the state. Each green flag represents an individual CalNat program partner, with numbers representing the partners in alphabetical order. Full program partner listing can be found at http://calnat.ucanr.edu/Take_a_class/

The mission of the California Naturalist program is “to foster a diverse community of naturalists and promote stewardship of California's natural resources through education and service” (UCANR, 2020a). Since its inception in 2012, CalNat has offered certification courses through 56 partner organizations operating in 51 of California's 58 counties (Figure 1). CalNat courses have certified over 3,700 naturalists who have logged over 168,000 hr of volunteer service, contributing over $5 million worth of environmental stewardship to the state of California (UCANR, 2018). Every CalNat course shares a core curriculum covering natural history topics in California, and every student completes the same requirements in order to receive certification. Individual courses may differ in terms of timing and structure, their bioregion or habitat of focus, and the range of instructional delivery methods they employ. Classes are generally small, ranging in size from 10 to 30, in order to support the focus on experiential, field‐based, hands‐on learning. Field activities are a required component of the course and are designed to develop skills such as making observations (using one's own senses and field equipment), drawing inferences using prior knowledge, and making measurements using common field equipment and protocols. These skills are cultivated through group field trips, participatory science projects, interpretive walks, homework assignments, and capstone projects that combine service and learning. In keeping with the program's central mission, research on CalNat courses and naturalists has shown that completing a CalNat course increases content knowledge on California ecosystems and environmental issues, raises student confidence in addressing environmental issues, and inspires long‐term engagement with participatory science and environmental stewardship (Merenlender et al., 2016).

The Wild Davis CalNat course was scheduled to begin at 1 April 2020; Yolo County and the UCD campus released shelter‐in‐place directives on 18 March 2020. Delaying the Wild Davis start date was not possible, given that the course is tied to the UCD academic calendar. Additionally, canceling the course could have had negative ramifications for student credit hours in terms of full‐time student status, financial aid, and spring graduation for seniors. Consequently, the authors of this paper, who represent the Wild Davis instructors, the Central Valley CalNat Community Education Specialist, and the CalNat Director, opted to restructure the course components to meet both the instructional expectations of the UCD campus and the core certification requirements of the CalNat program via remote instruction. This paper outlines the components of traditional instruction in this course (as implemented in the 2019 Wild Davis cohort), the process by which field‐based components were transitioned to remote instruction using participatory science projects (as implemented in the 2020 Wild Davis cohort), and offers guidance for faculty facing similar decisions in ecology and environmental science field courses.

This paper will use the term ‘participatory science’ to refer to publicly available programs and platforms through which amateur and nonprofessional scientists engage in scientific research through question development, study design, data collection, and analysis. While terms such as ‘citizen science’ are often used to describe these programs, the political connotations suggested by the word ‘citizen’ are at odds with the open and collaborative nature of these programs and have inspired careful consideration of the terminology (Eitzel et al., 2017). While various terms (e.g., ‘community science’) reflect the variety in training, purpose, geographic scope, and number of participants that these projects employ, none of them is U.S. citizenship necessary or relevant. Consequently, the authors of this paper follow the CalNat program's commitment to inclusive language (Angulo, 2020) that honors the diversity of California Naturalists and California residents, and credits their role in public science initiatives.

Participatory science takes many forms in CalNat courses. Students are required to log observations on iNaturalist (discussed in the iNaturalist Project section) and participate in an additional instructor‐chosen participatory science project (discussed in the Participatory Science Projects section). In addition, many students’ capstone projects focus on or relate to participatory science (discussed in the Capstone Projects section), and under remote instruction, many students chose to complete their field activities at their sit spot (discussed in the Nature Journaling section), which under traditional instruction is reserved for solo observation activities.

## 2 IRB/CAMPUS REVIEW STATEMENT

This case study was determined to be exempt from the UC Davis Institutional Review Board review process based on U.S. Department of Health and Human Services guidelines. It falls under the Quality Assurance/Quality Improvement activities exception as it pertains to assessing or improving a program (in this case, the course) and is not experimental in nature. Additionally, the manuscript was reviewed by representatives of the following campus offices to ensure compliance with student data and privacy guidelines: Center for Educational Effectiveness, Office of Information and Educational Technology, and the University Registrar (the campus FERPA officer).

## 3 TRANSITIONING TO REMOTE INSTRUCTION

All CalNat courses share core curriculum, structure, and requirements, though each course differs slightly in its content focus and delivery. This section outlines the core components of CalNat courses, including a description of their structure and educational value, and their implementation in Wild Davis under in‐person and remote instruction. In each section, specific comparisons are made between the 2019 Wild Davis cohort (which occurred in spring quarter 2019 under traditional in‐person instruction) and the 2020 Wild Davis cohort (which occurred in spring quarter 2020 under remote/online instruction).

### 3.1 Contact hours, readings, and general course structure

#### 3.1.1 Description and educational value

Students are required to attend all class sessions and field days (one absence is permitted with makeup work) which must total 40 hr throughout the course. The combination of lecture‐style instruction and hands‐on activities allows students to connect and apply content presented in the classroom to conditions in the field (Gleeson et al., 2012). Additionally, students must read the course textbook, *The California Naturalist Handbook* (de Nevers et al., 2013), in its entirety, in addition to any relevant bioregional or topical publication as part of the UCANR 8,000 online publication series (UCANR, 2020b), and complete assigned homework. The *Handbook* covers information on California habitats, communities, and environmental issues across the state, while the UCANR 8,000 publication series provides optional, focused content to deepen student exposure to particular bioregions. The use of a shared central text ensures consistency of content delivery across CalNat courses and provides a content framework around which the lecture content can be organized, and to which field activities can be linked.

#### 3.1.2 Traditional and remote implementation

Since UCD is on the quarter system, Wild Davis is structured as a four‐credit, 10‐week course offered during the spring quarter, enrolling 25–30 students. Under traditional instruction, the class meets once a week for three hours during which approximately an hour is spent in lecture‐style content delivery and explanation of the week's project (totaling ~10 contact hours), and the remainder of the class time is spent ‘in the field’ performing urban ecology data collection and experiments across campus (totaling ~20 contact hours). External activities account for the remainder of the contact hours (example: nature journaling activities) which students completed asynchronously and individually.

Under remote instruction, lecture‐style instructional hours were shifted to synchronous Zoom lectures (still accounting for ~10 total hours), with the goal being that synchronous content would provide opportunities to support the development of social relationships in the course. The social dimensions of learning are often underemphasized in teaching and learning, yet educational research consistently shows how social relationships are an important mediator of educational processes and outcomes (Vygotsky, 1978). The remaining 30 hr were completed by students asynchronously and included completion of the weekly ‘group’ field project (~20 total hours, see Participatory Science Projects Section for details) and solo field projects (~10 total hours, see Nature Journaling and Other Course Components sections for details). Zoom lectures were recorded and posted to the course Learning Management System (LMS, in this case Canvas) for student reference. Course points were awarded for attendance; however, students who could not attend live lectures could make up these points. Learning objectives for each lecture were rephrased as questions, and a student could provide written responses to the questions in order to receive ‘attendance’ points for that lecture. Even with this makeup option in place, students consistently attended the lectures in person with the result that the makeup option was only requested once throughout the entire quarter.

Under traditional instruction, students purchased *The California Naturalist Handbook* from the campus bookstore, or accessed a copy on reserve at the campus library. During remote instruction, students could also access an e‐text of the book, distributed by the campus library at no charge. In both quarters, supplemental readings were posted to the LMS at no charge to students.

### 3.2 Nature journaling

#### 3.2.1 Description and educational value

Throughout the course, each student creates a nature journal following the Grinnell method (Herman, 1986) with entries from all field and observation activities. Nature journaling can be an effective pedagogical tool for promoting observation of the natural world and connecting with the nonhuman environment (Tsevreni, 2020). Nature journaling can be particularly relevant in urban settings, which are the focus of Wild Davis activities, for focusing students’ attention on the patchwork of natural and anthropogenic habitat that exists in urban settings (Warkentin, 2011). Nature journaling in Wild Davis takes a variety of forms, including notes from lectures and readings, data collection and documentation, field notes and descriptions of activities completed, and sketches of urban flora and fauna, as well as narratives of observation activities and experiences.

Many CalNat courses, including Wild Davis, promote nature journaling at ‘sit spots,’ which are student‐selected locations the students are expected to observe in depth throughout the course. In Wild Davis, students visit their sit spot three times throughout the quarter, once around dawn, once at mid‐day, and once around dusk, for 45 min each. Sit spots are intended to be a solitary experience for the students, excluding friends, family, and electronic devices. Students practice focusing on each sense individually (with the exception of taste) to ‘get to know’ their location, and document their experience in words, sound recordings, sketches, and a limited number of photographs (to reduce device use). This provides an avenue for place‐based learning, or learning about “local natural, built, and social environments through inquiry, environmental action, and other hands‐on activities in a specific place” (Kudryavstev et al., 2012, p. 240). In addition to fostering pro‐environmental behavior, place‐based education can foster academic achievement, positive social–emotional outcomes, and greater appreciation of the natural world (Sobel, 2005). Participation in direct instruction through synchronous lectures, in combination with engagement in multiple forms of experiential activity such as nature journaling, observation activities, and completion of field activities, represents a rigorous and effective strategy to strengthen students’ sense of place (Kudryavstev et al., 2012).

#### 3.2.2 Traditional and remote implementation

Under traditional instruction, nature journals were used by students for taking notes on lecture content, documenting their contribution to the group field projects (including data collection), and narrating and sketching their experiences at their sit spots. The 2019 cohort used their sit spots exclusively for solo nature journaling and observation activities. Students were encouraged to choose sit spots in an urban green space, garden, or park. Since the sit spot observations occur at times of day when students may not be comfortable being alone outdoors, any space in which the student felt safe was allowed (including backyards). After each observation, students turned in their nature journals for review by the instructor.

Under remote instruction, the 2020 cohort students were even more explicitly encouraged to choose a sit spot in which they felt safe, and in which prolonged sitting was allowed. Under shelter‐in‐place directives, many city parks required visitors to be exercising and not ‘loitering’ which limited the options for students in 2020. Consequently, several students selected sit spots in their backyard (*n* = 9/23), though many still chose a neighborhood park or greenspace (*n* = 14/23). The 2020 cohort was also allowed to change the location of their sit spot if the student's access to or comfort with the location changed as a result of the COVID pandemic. Students completed their observations in their nature journals and then ‘turned in’ their journal entries via scans/photographs or typed transcripts of the journal pages uploaded to the course LMS. The 2020 cohort of students often used their sit spot to also complete other course activities (described in the Participatory Science Projects section) expanding on the place‐based learning component of these activities.

### 3.3 iNaturalist project

#### 3.3.1 Description and educational value

iNaturalist is an online participatory science portal in which members of the public upload photos or audio recordings of wildlife. Observations are tagged with location and time/date stamps and publicly searchable on the organization's website (inaturalist.org). Identification of organisms is assisted with an artificial intelligence (AI) interface which suggests possible taxonomic groups, as well as a robust community of users who suggest identifications and/or confirm other user's suggestions. Photographs or audio recordings can be taken and uploaded in the moment on a mobile device, or with a traditional camera and uploaded later from a laptop or desktop.

All certified California Naturalists are expected to be familiar with iNaturalist and have logged at least 1 observation in the app (in addition to the class participatory science project, discussed in the next section). iNaturalist activity introduces students to a robust community of naturalists and encourages long‐term activity in practicing naturalist activities and identification (Unger et al., 2020). iNaturalist's AI species identification support helps students build and test their naturalist skills on their own time and at their own pace, whenever and wherever they are able to photograph an organism of interest.

#### 3.3.2 Traditional and remote implementation

Wild Davis students participate in the iNaturalist‐focused City Nature Challenge (CNC, citynaturechallenge.org) which promotes iNaturalist observations of urban wildlife in cities around the world during a four‐day weekend. The 2019 cohort performed a practice ‘bioblitz’ (documentation of as many organisms as possible in a given area) on campus together during class to practice interacting with the app and finding organisms. Students then participated in the Sacramento Region City Nature Challenge outside of class time from 26 to 29 April 2019. Students were required to post 20 observations of wild organisms (not captive or cultivated) within the nine‐county Sacramento Region (which includes the town of Davis and the UCD campus) during the CNC window.

The 2020 CNC was heavily impacted by shelter‐in‐place guidelines and limited access to regional greenspaces. Numerous participating cities opted to cancel their CNC entirely. Other cities canceled all formal gatherings and events, and transitioned to a “City Nature Celebration,” reducing the competitive focus of the CNC and emphasizing observing backyard wildlife. The majority of the 2020 cohort (21/23) resided in a CNC‐participating region, and students outside these regions could still post observations to iNaturalist during the CNC window, though their observations were not counted in CNC totals. As in 2019, students in the 2020 cohort were required to post 20 observations; however, to accommodate shelter‐in‐place directives in 2020, cultivated plants (including landscaping and gardens) were allowed, so long as the observation was marked “Captive/Cultivated” on iNaturalist.

**FIGURE 2:**
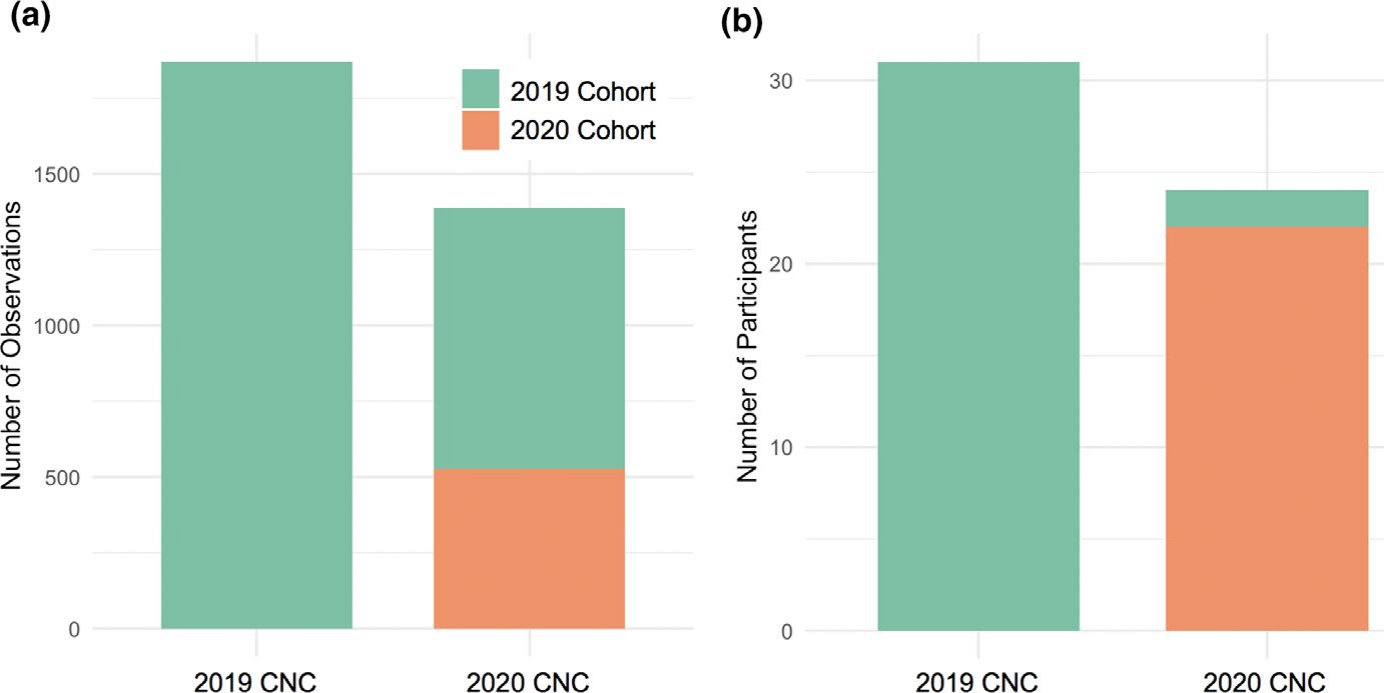
Wild Davis participation in the 2019 and 2020 City Nature Challenges (CNCs). Panel a shows the number of observations in each CNC year by each Wild Davis cohort; Panel b shows the number of participants in each CNC by Wild Davis cohort. Since enrolled students were required to participate in the CNC as part of the course, counts in Panel b reflect full enrollment in the course. Colors represent students enrolled in Wild Davis in Spring Quarter 2019 (green, traditional instruction) and Spring Quarter 2020 (orange, remote instruction)

Figure 2 compares Wild Davis students’ CNC participation between 2019 and 2020 in both numbers of observations (Figure 2a) and number of participants (Figure 2b). Since participation was required in both quarters, percent of participating students was 100% in both quarters. Likely due to limitations to outdoor recreation under shelter‐in‐place guidelines, overall Wild Davis participation in the CNC was reduced in 2020 compared with 2019 (Figure 2a). Prior to the spring 2020 quarter, only one 2020 Wild Davis student had an iNaturalist account and no 2020 students participated in the 2019 CNC (Figure 2b). Interestingly, the majority of 2020 CNC observations by Wild Davis students were made by students from the 2019 cohort (862/1389, 62%, Figure 2a), though this large contribution was generated by a small number (*n* = 2) of individual students (Figure 2b). These data support the finding of Merenlender et al. (2016) that students’ engagement with participatory science projects continues even after the completion of their CalNat course.

### 3.4 Participatory science projects

#### 3.4.1 Description and educational value

In addition to logging observations on iNaturalist, each CalNat course chooses an additional participatory science project to highlight in the class. Coupled with the iNaturalist activity, this requirement exposes students to the wide range of participatory science endeavors available to the public and the students’ ability to meaningfully contribute as a cohort to a scientific endeavor following established protocols. CalNat course content lends itself rather easily to participatory science projects. For example, one chapter of the *Handbook* broadly covers California's animal communities, including trophic interactions, functional groups, introduced and invasive animals, and highlights of interesting or iconic California wildlife in a variety of taxonomic groups. Participatory science projects that could be integrated into this content include (but are not limited to):

● iNaturalist: through either general wildlife observations or with respect to specific projects tracking individual taxa or regions, including invasive species.
● Taxon‐specific observation projects: eBird, Bumble Bee Watch, Bat Detective (through SciStarter), etc.
● Season specific projects: Nature's Notebook (through the National Phenology Network), NestWatch, etc. Courses operating in the fall could also take advantage of seasonal projects such as the Christmas Day Bird Count (through the Audubon Society).
● Region specific projects: UCD Tricolor Blackbird Project, Western Monarch Milkweed Mapper, etc.
● Abiotic projects with a conceptual link to wild‐life issues: GLOBE at Night (light pollution), Debris Tracker (litter and garbage), Stream Selfie (through SciStarter; water quality and flow patterns), etc. Note that projects of this nature would require more framing and perhaps additional research outside of the participatory science program in order to strengthen links to course content.

In Wild Davis, each week of coursework includes content from one chapter of *The California Naturalist Handbook* and a field activity aligning with that week's content. In both traditional and remote instruction, several of these field activities were participatory science projects, though the specific projects and the nature of the students’ activity with them varied between traditional and remote instruction (Table 1).

**TABLE 1:** Participatory science projects incorporated into Wild Davis under traditional and remote implementation

Name of project	Description of activity	Used with remote instruction	Particular sampling supplies	Particular sampling protocol	Requires phone or computer	Reported data easily accessible	Assists with identification
iNaturalist	Upload and identify photos or audio of wild organisms	X			X	X	X
School of Ants	Capture ants with cookie bait, mail to program for identification		X	X			
CALeDNA	Collect soil or sediment, mail to program for environmental DNA analysis		X	X	X	X	
The Great Sunflower Project	Count and identify pollinators on plants	X		X	X^a^	X^b^	X
Bumble Bee Watch	Upload and identify photographs of bumble bees	X			X	X	X
GLOBE Clouds	Photograph and identify clouds and contrails	X			X	X^c^	X
GLOBE at Night	Document visibility of stars and constellations in urban areas	X			X	X	X
Debris Tracker	Document quantity and type of litter and garbage while cleaning up a site	X			X	X	

^a^ The Great Sunflower Project datasheet could be completed on paper and mailed to the instructor for submission to the web portal.

^b^ The Great Sunflower Project posts maps of observation locations and graphs of total numbers of observations each year, but details of observations are not visible or searchable.

^c^ GLOBE Cloud observer data are searchable/visible on the mobile app, and some observations can be downloaded from the web portal.

#### 3.4.2 Traditional and remote implementation

Under traditional instruction, the 2019 cohort completed three participatory science projects. All of these projects were completed together, on campus, during regularly scheduled class time. Other field activities focused on urban ecology observation activities not linked to a participatory science projects. Under remote instruction, online participatory science projects made up half of the field activities (4/8). The remaining activities included online research projects (2/8) and ecological observation activities not linked to an established participatory science project (2/8), which are discussed in the Other Course Components section. Under remote instruction, the focus of the field activities relied more heavily on the structure of the participatory science project, whereas traditional instruction often included sampling for such projects as one part of a multi‐faceted field experience. Consequently, while participatory science projects were only slightly more numerous under remote instruction than traditional instruction, they made up a much larger proportion of the focus and goals of the field activities.

The participatory science projects used in Wild Davis in 2019 and 2020 are listed in Table 1, along with some important traits for consideration when choosing a project. Under remote instruction, the most important traits were that the students could complete the activity from any location (especially a backyard) without special sampling equipment or training, and with limited guidance from the instructor. The lack of in‐the‐moment, one‐on‐one guidance led the instructor to select projects that assisted users with identifying the organisms or structures in their observations. Additionally, the constraint of using backyard or neighborhood locations led the instructor to select projects whose website allowed for viewing and visualizing previously reported observations. In this way, even if a student's backyard was not conducive to making new observations in the project, the student could still participate in the activity by summarizing other user's observations from their region or comparing nearby regions. This secondary option for participating in the field activities was only necessary for the Debris Tracker activity, in which not all students were able to find litter or debris in their neighborhood.

Two participatory science projects were used in traditional instruction in Wild Davis in 2019 but were not used in 2020: CALeDNA and School of Ants (Table 1). Both projects have a particular sampling protocol to follow and require some sampling and collection supplies. While the sampling protocol is simple and could easily be completed in a backyard, the short timeline of the transition to remote instruction precluded mailing students the necessary supplies. Additionally, when completed in traditional instruction, these field activities included guest speakers who shared their personal involvement with the project, supported in‐the‐moment identification, and/or led a detailed naturalist tour of the sampling area. The instructional team believed the lack of these components undermined the experiential value of the project and replaced these activities in remote instruction.

In both traditional and remote instruction, students received course points for completing the weekly field activities. Under traditional instruction, the instructional team observed the 2019 cohort students completing the activity in real time. Under remote instruction, students needed to provide ‘evidence’ of their participation in the project. In most cases, this took the form of uploading a screenshot of their submission to the participatory science project and a short description of how and where they collected their data to the course LMS.

In the majority of cases, the instructor contributed data or observations to the project prior to the course session in which the activity was introduced and could then share their data/observations as an example. Interacting with the data submission portal prior to assigning the activity was also important for troubleshooting problems the students might encounter in completing the activity. For example, GLOBE Cloud Observer requires submitted observations to be reviewed before they are visible in the public data record. This extra step meant that students needed to either (1) complete the activity at least a day before the assignment deadline to ensure their observations were visible to the instructor or (2) take a screenshot of their observation prior to submitting it to the portal, which they could share with the instructor to document their activity.

User privacy is also an important consideration when requiring students to participate in online activities. The majority of the participatory science projects used in this class (Table 1) allow for anonymous reporting. Even when the project posts maps or visualizations of observations, the user who submitted the data is not always reported (e.g., GLOBE Cloud Observer). Even projects that report the observer's name allow for users to use a screen name that need not be their legal name and report no other identifying information (e.g., Debris Tracker and iNaturalist). The most robust privacy measures, illustrated by iNaturalist, allow users to obscure the geolocation of their observation, which is particularly important when students are primarily making observations in or near their home. Additionally, the option of summarizing or analyzing other user's data could be used as an option for students uncomfortable with the required online presence of participatory science projects. Indeed, research has shown that engaging with the broader dataset in which their individual observations are ‘nested’ has a positive impact on student valuation of participating in such projects and on students’ perception of themselves as agents of environmental change (Harris et al., 2020).

### 3.5 Capstone projects

#### 3.5.1 Description and educational value

CalNat capstone projects require no more than eight hours of outside of class service learning in support of an environmental or nature education organization. Capstone projects must fall into one of six categories: environmental stewardship or habitat restoration, education or interpretation, program support, climate and environmental justice, community resilience and adaptation, or participatory science. Through capstone projects, students demonstrate competence in a specific topic through the design, initiation, and completion of an environmental service project in support of a local education, conservation, participatory science, or program support initiative. Capstone projects serve two educational goals: (1) incorporating student‐driven course content, which has been shown to increase student motivation toward and engagement with fieldwork (Pawson & Teather, 2002), and (2) promoting long‐term activity in the CalNat community by exposing students to volunteerism and community service opportunities.

#### 3.5.2 Traditional and remote implementation

Under traditional in‐person instruction, capstone projects were a mix of data collection for management purposes (e.g., water quality measurements in the UCD Arboretum and Public Garden waterway), infographics and public signage (e.g., infographic on data collected by the Lake Tahoe Environmental Research Center's participatory science project Snapshot Day), and hands‐on product development (e.g., bee nesting post for the Davis Central Park Pollinator Garden). Under remote instruction, capstone projects needed to be coordinated and completed solo and exclusively online. These restrictions meant that the majority of capstone projects were interpretive resources such as infographics and guides (e.g., raptor ID guide for the UCD Raptor Center, https://crc.vetmed.ucdavis.edu/) or educational activity books for elementary age students (e.g., Take Care Tahoe activity booklet; https://takecaretahoe.org/) which could be disseminated online. Additionally, several students used the participatory science project GLOBE at Night to track patterns in urban light pollution. This project was not formally used as a group activity in the course, but worked well for capstone projects given its focus on urban observations and so is included in Table 1.

Two capstone projects related directly to the COVID pandemic. One of these involved a “Truth About Bats” infographic developed in conjunction with the Yolo Basin Foundation (http://yolobasin.org/) to counter misinformation on the relationship between bats and disease. A second capstone project focused on mapping the location of CNC observations in urban centers to compare the frequency of observations in urban greenspaces versus residential locations in the 2019 CNC versus the 2020 CNC. This project was developed in conjunction with the UC Davis Center for Community and Citizen Science (CCCS), an organizer for the Sacramento Region CNC, and was highlighted on their public newsletter (Monty, 2020). This capstone project led to an ongoing collaboration even after the official end of the course between this Wild Davis student and CCCS surrounding mapping of other participatory science projects and activities with which CCCS is involved.

Students in both the 2019 and 2020 cohorts also presented the final product of their capstone project with the class, which took the place of a final exam. Since the capstone projects are completed mostly on the students’ own outside of class time, this presentation served to illustrate to the group the type of capstone project each student completed and give each student a chance to share the results of their hard work with the group.

### 3.6 Other course components

#### 3.6.1 Description and educational value

The Wild Davis course incorporates two components that have not been covered in the previous sections. (1) Field activities not associated with a participatory science project and (2) group presentations of field activities by students. Field activities without a participatory science project focus contribute to variety in the student experience and course activities. Group presentations provide the students a chance to share their experiences with the class and/or report summary data on class‐wide observations. Each week, a group of 2–4 students report on the outcomes or experiences of the previous weeks’ activity. Students sign up for the topic of their choice and can work together as a group, or present individually.

#### 3.6.2 Traditional and remote implementation

In order to provide some variety to students on their weekly activities, four of the eight field projects did not incorporate online participatory science projects. Two of these were short online research projects asking students to research a Superfund site (https://www.epa.gov/superfund) or land trust (e.g., California Council of Land Trusts; https://www.calandtrusts.org/) active in their hometown or place of residence. Two other activities involved outdoor field observations not linked to a participatory science project. One of these was an urban ecology scavenger hunt in which students collected photographs of ecological interactions in their backyard or neighborhood (e.g., an interaction between an animal and a plant; a mutualistic interaction, etc.). Many students completed this activity without leaving their yard; indeed, the smaller spatial scale inspired additional creativity for some of the more uncommon scavenger hunt items. The second of these activities was an audio identification activity for local birds. This activity was developed specifically because several students had commented in their first sit spot observation that they heard birds they could not identify. The instructor brought in a guest speaker who has experience in teaching ‘birding by ear’ to help students practice describing bird calls and identifying common birds solely by their sounds. The activity for the week then focused on audio recordings from the students’ sit spot in which they identified bird calls, other biotic sounds (e.g., squirrels), and anthropogenic sounds (e.g., cars, construction).

Each class session began with a group presentation over the previous week's activity. Under traditional instruction, these presentations were done via the classroom audiovisual setup and focused on summarizing the class data or observations. Under remote instruction, group presentations occurred via Zoom; students needed to unmute and speak to the group, but did not need to turn on their webcams. Students whose Internet bandwidth was not reliably able to screenshare sent their presentation to the instructor who shared it for them. Given the asynchronous structure of the activities under remote instruction, most group presentations focused on the students’ own contributions to the activity, since students were not completing the activity at the same time together. Under both forms of instruction, participatory science projects with data reporting tools allowed students to compare observations or data generated by the class to previously reported data from the region.

## 4 STUDENT FEEDBACK AND ENGAGEMENT

As part of the course, Wild Davis students in both the 2019 traditional and 2020 remote instruction cohorts completed two surveys: a CalNat postcourse survey and a UCD student course evaluation. These surveys provide feedback on the student perspective of and experience in the course for long‐term evaluation and improvement of Wild Davis and the statewide CalNat program. In addition, both cohorts of students created a profile in the CalNat Volunteer Management System (VMS), in which they recorded volunteer service hours, starting with the time dedicated to completing their capstone project. Students were also asked to consider and hopefully commit to volunteering at least 40 hr a year in environmental stewardship, education, program support, climate and environmental justice, community resilience and adaptation, or participatory science and to record all hours of service in the VMS. The CalNat program communicates with the statewide naturalist community via the VMS, including announcements of volunteer needs or opportunities, and program networking events. CalNat also encourages continued volunteerism by awarding an annual pin to naturalists who record 40+ annual service hours. The VMS also provides the program with an estimate of continued engagement with naturalist activities and the impact of the CalNat community throughout the state.

While bias in student evaluations is well documented (Stark & Freishtat, 2014), many of the triggers of bias (such as those relating to instructor identity) were the same between the 2019 and 2020 cohorts; the most influential exceptions being response percentage (which was lower in 2020), and the slight change in wording of UCD campus evaluation questions (Table 2).

**TABLE 2:** Course survey and evaluation results from traditional (2019) and remote (2020) instruction

Category or Question	Spring 2019	Spring 2020
Overall rating of instructor^a^ (via UCD course evaluations)	4.8 ± 0.6 (max 5)	4.9 ± 0.3 (max 5)
Overall rating of course^b^ (via UCD course evaluations)	4.7 ± 0.8 (max 5)	4.9 ± 0.3 (max 5)
Overall Satisfaction^c^ (via CalNat course survey, 2018 state average 92%)	96%	92%
Instructor Performance^d^ (via CalNat course survey, 2018 state average: 91%)	100%	100%
Plan to Volunteer^e^(via CalNat course survey)	80%	92%
Self‐confidence, pre/post^f^ (via CalNat course survey, 2018 state average: 26/47%)	17/50%	62/100%
Response rate, UCD/CalNat	94/79%	73/54%

^a^ In the spring 2019 evaluation, this question was worded “What is your overall evaluation of the instructor: excellent (5) | above average (4) | average (3) | below average (2) | poor (1)” while in the Spring 2020 evaluation, this question was worded “Please indicate the overall teaching effectiveness of the instructor: excellent (5) | very good (4) | satisfactory (3) | fair (2) | poor (1)”.

^b^ In the spring 2019 evaluation, this question was worded “What is your overall evaluation of the course: excellent (5) | above average (4) | average (3) | below average (2) | poor (1)” while in the Spring 2020 evaluation, this question was worded “Please indicate the overall educational value of the course: excellent (5) | very good (4) | satisfactory (3) | fair (2) | poor (1)”.

^c^ Percent of respondents that responded ‘satisfied’ or ‘very satisfied’ to the prompt “Overall, how satisfied were you with the California Naturalist course you attended? Very satisfied | Satisfied | Somewhat Satisfied | Somewhat Unsatisfied | Not Satisfied”.

^d^ Percent of respondents that responded ‘very good’ or ‘excellent’ to the prompt “How would you rate the performance of your lead instructor? Excellent | Very Good | Good | Needs Improvement | Poor”.

^e^ Percent of respondents that responded ‘Yes’ to the prompt “As a California Naturalist, do you plan to volunteer in the coming year? Yes | No”.

^f^ Retrospective pre/postcourse; percent of respondents that ‘strongly agree’ they are “capable of making a positive impact on the environment”.

**FIGURE 3:**
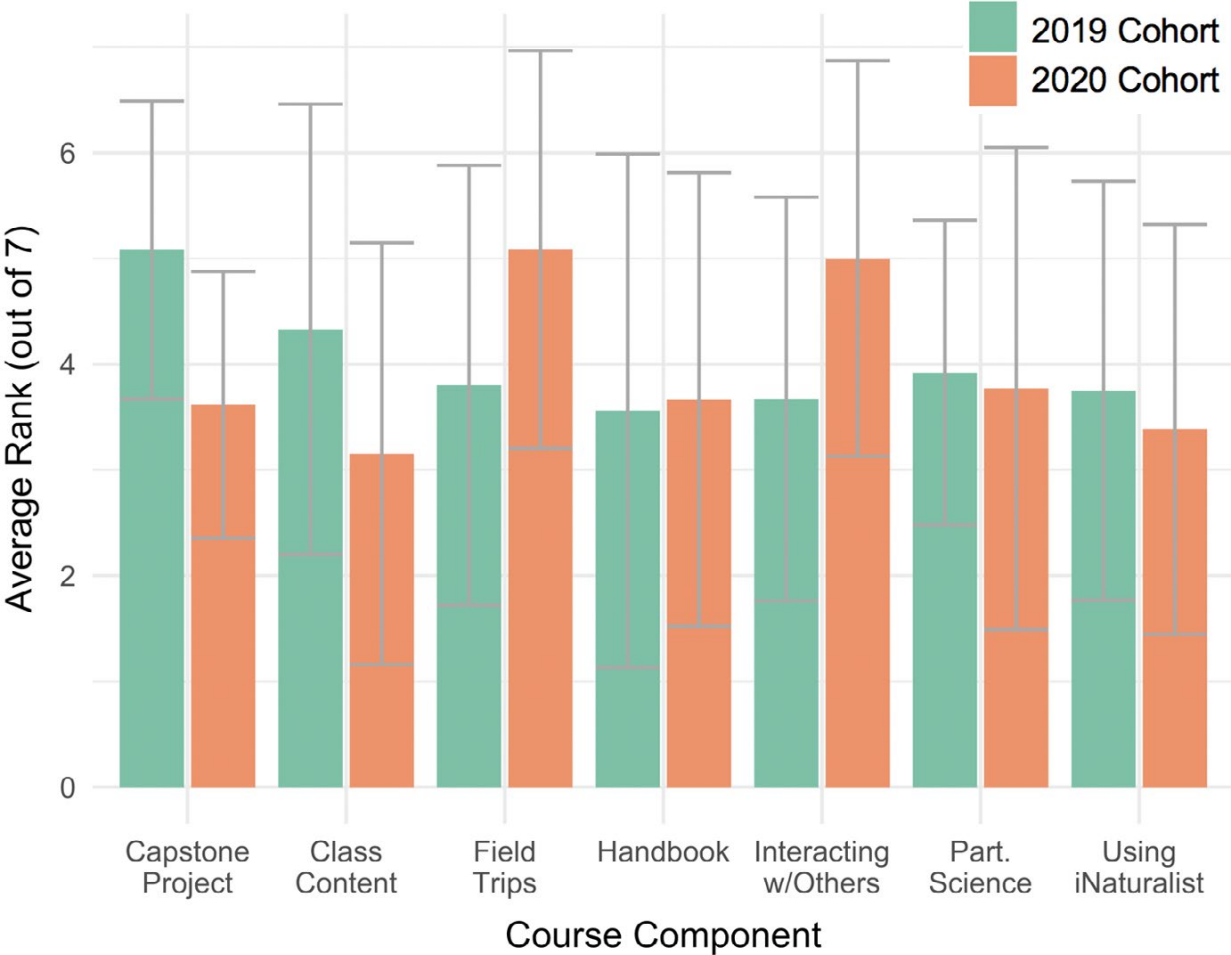
Student ranking of various course components on anonymous course evaluations in 2019 (green) and 2020 (orange). Gray bars represent standard deviations

Anonymous end‐of‐course student evaluations indicate the 2020 remote instruction experience was on par with the 2019 traditional instruction experience in terms of student engagement (Table 2, Figure 3). Generally, student perspectives on the course were highly positive and the transition to remote instruction resulted in minimal change in overall ratings (Table 2). When asked on the evaluations to rank individual components of the course as most to least helpful, the 2020 cohort identified field trips and interacting with others in the class as the most beneficial components (Figure 3). Interestingly, these two components were ranked higher in the remote 2020 course than the traditional in‐person 2019 course. The survey results are consistent with anecdotal evidence from student–instructor conversations. Most commonly in these conversations, students appreciated synchronous lectures, as many of their classes were fully asynchronous and provided little opportunity for interaction. Additionally, students appreciated the opportunity, indeed the requirement, to leave their computer screen and go outside even only to their backyard for a short while. As previously mentioned, social relationships (Vygotsky, 1978) and a sense of place (Kudryavtsev et al., 2012) are important components of the educational environment, perhaps even more so during COVID when students were quarantined indoors and isolated from the majority of their interpersonal interactions. These results promisingly indicate that it is possible to develop engaging field components and interpersonal interaction under remote instruction and shelter‐in‐place directives.

The most diverging evaluation statistic between the two course offerings was the pre/postquestion on self‐confidence (Table 2). The 2019 cohort entered with very low confidence and ended near the state average for participants in other CalNat courses, while the 2020 cohort entered with higher confidence and ended extremely high. It is difficult to interpret these numbers. The unusually high 2020 precourse values may be a result of students self‐selecting, if the course attracted students who were already aware of and educated in California environmental issues, though why this would be the case in 2020 and not 2019 is unclear. The unusually high 2020 postcourse values, and the unusually high gain in self‐confidence from precourse to postcourse, may also be related to the higher emphasis on interacting with other user data in participatory science projects. As mentioned previously, interacting with the broader dataset in which their observations are nested has been shown to have a positive impact on students’ perception of themselves as agents of environmental change (Harris et al., 2020). Additionally, the 2020 values may have been disproportionately impacted by the low response rate to the CalNat survey and not reflective of the 2020 cohort as a whole.

**FIGURE 4:**
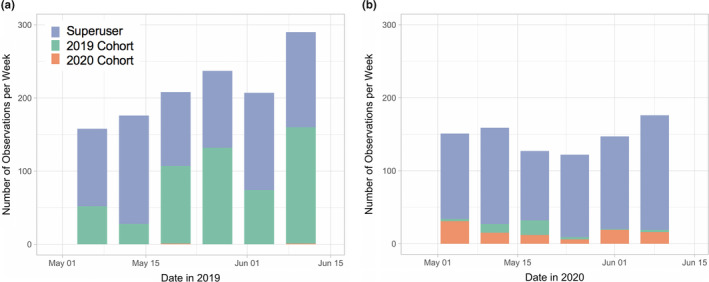
Continued activity on iNaturalist following the CNC in 2019 (a) and 2020 (b), with colors representing the 2019 Wild Davis cohort (green), the 2020 Wild Davis cohort (orange), and the individual superuser from the 2019 Wild Davis cohort (blue)

Another metric of student engagement relates to continued volunteerism and service, recorded in the VMS, and continued activity in the participatory science projects introduced in the course. Continued shelter‐in‐place and social distancing directives have severely limited the service and volunteerism opportunities, resulting in only one student from either cohort having recorded service hours during the summer of 2020. iNaturalist provides the clearest picture of participatory science project activity, given its broad focus in terms of taxonomy and geography, its robust data access portal, and the fact that it is the most user‐friendly participatory science project in the course, and the one with which the students interact the most. Figure 4 shows the number of observations students in the 2019 traditional instruction and 2020 remote instruction cohorts contributed to iNaturalist in the six weeks following the end of the CNC (when required activity on iNaturalist as part of the course was complete). In both years, students remained active in iNaturalist following the completion of the CNC. In both years, the majority of observations were contributed by a single ‘superuser’ student in the 2019 cohort whose interest in iNaturalist was unusually high. Interestingly, while activity on iNaturalist by 2020 Wild Davis students following the 2020 CNC was much lower than that of the 2019 cohort following the 2019 CNC, it was still nonzero and maintained for the full six‐week window despite continued shelter‐in‐place directives. These results suggest that for some students, engagement with and enjoyment of participatory science continued beyond the class project and became a part of their regular activities. These results are also consistent with a resurgence in interest in community and backyard gardening during COVID (while no formal data yet exist to our knowledge, see Walljasper & Polansek, 2020).

In this section, we compared student engagement in and evaluation of the fully remote 2020 offering and the traditional in‐person 2019 offering of the course. We recognize that, had the students experienced both versions of the course, the remote 2020 offering may not have rated so highly. It is worth noting, however, that the majority of students experience a course only once and so cannot compare it to previous or subsequent offerings (as instructors can). These results indicate that students responded positively to a remotely instructed field course that was as engaging as possible, even if it may still have been less engaging than the course was in person.

## 5 FUTURE WILD DAVIS AND CALNAT COURSES

Given the focus of the course on field‐based, experiential learning, many remote instruction components such as asynchronous field activities and online research projects may not be continued in future traditional instruction iterations of this course. Even so, these components could contribute to future course development. For instance, the online research projects served to identify possible future collaborators for student capstone projects, such as local land trust organizations. Similarly, the ‘birding by ear’ activity was popular with students, likely due, in part, to the activity being developed specifically in response to student‐driven interests and desires (Pawson & Teather, 2002). While the activity was not linked with a participatory science project, it could have been, as both eBird and iNaturalist allow audio file observation submissions. This activity will be continued in future iterations of the course, and the instructors plan to offer explicit opportunities for students to suggest naturalist skills they would like to develop, around which course activities can be developed. The future goal of the instructional team is that all field activities in Wild Davis will incorporate a participatory science project. While remote instruction forced a rapid transition to a wider variety of projects and a stronger focus on the student experience within the project, this transition was in keeping with the long‐term goals for the course.

The results of Wild Davis' additions and alterations for remote delivery of the CalNat course curriculum have heavily influenced some of the future statewide program goals, particularly surrounding enhanced integration of participatory science into CalNat instruction. Consequently, an additional product being developed out of the 2020 remote instruction experience is a reference book for CalNat instructors on (1) participatory science projects that CalNat courses have implemented, (2) best practices or considerations for incorporating particular projects, and (3) a living collection of participatory science projects that align with CalNat goals and content focus.

## 6 CONSIDERATIONS FOR INTEGRATING PARTICIPATORY SCIENCE INTO FIELD COURSES

Faculty wishing to replace in‐person field activities with pre‐existing, online participatory science projects may wish to consider the following when selecting and integrating a project. The questions listed below are drawn from conversations and experiences the instructional team had as they developed and enacted activities under remote instruction. While the relevance of each consideration may vary by course content, structure, and logistics, the instructional team found that considering these points greatly aided in selecting and integrating participatory science projects into the course.

In terms of the logistics of the participatory science project:

● What experience, if any, do the students already have with the project? Students who have experience with the project will likely need less instructor guidance and support and may be able to serve as mentors or group project leaders for students with less experience.
● Does the project require particular sampling equipment? If so, this project may be difficult for students to complete if they do not have access to the equipment. The instructor may be able to mail students’ equipment, depending on the timeline and size of the class.
● Does the project have a complex sampling protocol? If so, it may be difficult for the students to follow the protocol accurately without one‐on‐one, real‐time guidance.
● Does the project support data quality via guidance or identification of organisms, structures, or processes being observed? If so, the students may find it easier to gather reliable data without one‐on‐one, real‐time guidance.
● Does the project require a mobile app for data submission? If so, students without a data plan may have difficulty participating. Can a computer or paper submission be used instead? If so, this makes the project more accessible to a wider cohort of students.
● Does the project provide user‐friendly access to reported data? If so, students may be able to complete a data summary activity instead of, or in addition to, data submission. Additionally, the instructor may be able to more easily track student participation in the project.
● Does the project provide an opportunity for interaction among participants? If so, the project could support the building of a sense of community in the course. For example, iNaturalist allows observers to comment on one another's observations and to create projects for individual taxa, regions, or observers. A course‐focused project allows students to easily see and comment on their colleagues’ observations.
● What is the expected duration of active participation in the project? Many projects allow for single‐observation activity (e.g., iNaturalist); however, prolonged or repeated observations may be expected or required (e.g., NestWatch). Either of these may be preferred, depending on how the project integrates into the coursework.

In terms of linking with course content and assignments:

● How does participating in the activity contribute to course learning goals and course content? What does the instructor expect the students to gain by participating in the project? Example goals include (1) learning to identify certain organisms or structures (e.g., clouds), (2) practicing scientific skills such as using sampling equipment or following a protocol, (3) developing data skills such as assessing data quality or repeatability, or visualizing data, and (4) understanding the process of science, such as generating and testing a hypothesis using data from the project, etc.
● How will students engage with the project and the data it generates? Examples include (1) submitting individual data/observations, (2) summarizing, analyzing, or visualizing other user's data from the project (either alone or in groups), and (3) comparing data from similar projects (e.g., particular species observations in iNaturalist versus eBird), etc.
● How will grades be assigned for the activity? How much participation in the project is expected for full credit? How will the instructor follow student activity or submissions in the project?
● What difficulties might the students experience in completing the activity? Are there situations in which a student may not be able to participate in the project? For this question, it is important for the instructor to test‐run making observations or contributing data to the project prior to assigning the activity to students.
● How does temporal and spatial variation in observations affect the outcomes of the activity? In some cases, such variation limits the ability to use the data in a meaningful way (e.g., due to low replication). In other cases, however, this variation can be useful. For example, under traditional instruction, making observations in GLOBE Cloud Observer together on campus would not make sense as all students would be observing the same clouds; however, spreading this activity across the geography of the students’ residences and over a week‐long time span allowed for greater variety in cloud types observed.

There are many additional resources to assist faculty in strategizing incorporating participatory science projects into a remotely instructed course, such as the Citizen Science Association's (https://www.citizenscience.org/) series of webinars on participatory science and higher education, all of which are available on the Citizen Science Association's YouTube Channel (https://www.youtube.com/channel/UChTgtIf9BqiEpWiczvH0jbA). Additionally, several ‘warehouse’ sites exist for accessing a variety of participatory science projects, which illustrate the diversity of projects available in terms of content focus and structure of participation. These include sites such as Zooniverse (https://www.zooniverse.org/), SciStarter (https://scistarter.org/), and CitSci.org (https://citsci.org/). SciStarter also recently released a field guide to participatory science projects geared toward a variety of age levels and content fields (Cavalier et al., 2020). Finally, a list of participatory science projects the CalNat program recommends and CalNat partner organizations use can be found on their website (http://calnat.ucanr.edu/California_PPSR/).

## 7 CONCLUSION

This case study illustrates how a field course focused on in‐person group projects relating to urban ecology was transitioned to fully remote instruction, primarily using pre‐existing online participatory science projects. While this strategy may not serve all field courses in terms of structure or content, the diversity of readily accessible participatory science projects is robust and continuing to increase. Additionally, the limited available evidence suggests that the remote offering of the course was similar to the traditional in‐person offering in terms of general engagement and student appreciation. Indeed, students valued interpersonal interaction and outdoor field components more highly in the remote instructed quarter than under traditional in‐person instruction.

## CONFLICT OF INTEREST

None declared.

## AUTHOR CONTRIBUTION

**Laci M. Gerhart:** Conceptualization (equal); Data curation (lead); Project administration (equal); Supervision (lead); Visualization (lead); Writing‐original draft (lead); Writing‐review & editing (equal). **Christopher C. Jadallah:** Conceptualization (equal); Project administration (equal); Writing‐review & editing (equal). **Sarah S. Angulo:** Conceptualization (equal); Writing‐review & editing (supporting). **Gregory C. Ira:** Conceptualization (equal); Writing‐review & editing (supporting).

## Data Availability

To ensure FERPA (Family Educational Rights and Privacy Act) compliance, the authors will share only aggregated, deidentified data upon request.
